# Rational selection of a biomarker panel targeting unmet clinical needs in kidney injury

**DOI:** 10.1186/s12014-021-09315-z

**Published:** 2021-02-22

**Authors:** T. T. van Duijl, D. Soonawala, J. W. de Fijter, L. R. Ruhaak, C. M. Cobbaert

**Affiliations:** 1 Department of Clinical Chemistry and Laboratory Medicine, Leiden University Medical Center, Postzone E2-P, Albinusdreef 2, 2333 ZA Leiden, The Netherlands; 2 Department of Nephrology, Leiden University Medical Center, Leiden, The Netherlands; 3 Department of Internal Medicine, Haga Teaching Hospital, The Hague, The Netherlands

**Keywords:** Acute kidney injury, Clinical needs, Biomarkers, Urine proteomics, Urinalysis

## Abstract

The pipeline of biomarker translation from bench to bedside is challenging and limited biomarkers have been adopted to routine clinical care. Ideally, biomarker research and development should be driven by unmet clinical needs in health care. To guide researchers, clinical chemists and clinicians in their biomarker research, the European Federation of Clinical Chemistry and Laboratory Medicine (EFLM) has developed a structured questionnaire in which the clinical gaps in current clinical pathways are identified and desirable performance specifications are predefined. In kidney injury, the high prevalence of the syndrome acute kidney injury (AKI) in the hospital setting has a significant impact on morbidity, patient survival and health care costs, but the use of biomarkers indicating early kidney injury in daily patient care remains limited. Routinely, medical labs measure serum creatinine, which is a functional biomarker, insensitive for detecting early kidney damage and cannot distinguish between renal and prerenal AKI. The perceived unmet clinical needs in kidney injury were identified through the EFLM questionnaire. Nephrologists within our tertiary care hospital emphasized that biomarkers are needed for (1) early diagnosis of in-hospital AKI after a medical insult and in critically ill patients, (2) risk stratification for kidney injury prior to a scheduled (elective) intervention, (3) kidney injury monitoring in patients scheduled to receive nephrotoxic medication and after kidney transplantation and (4) differentiation between prerenal AKI and structural kidney damage. The biomarker search and selection strategy resulted in a rational selection of an eleven-protein urinary panel for kidney injury that target these clinical needs. To assess the clinical utility of the proposed biomarker panel in kidney injury, a multiplexed LC–MS test is now in development for the intended translational research.

## Background

There is large potential for urinary biomarkers to improve patient care through early, noninvasive and precise diagnostics of early kidney injury. Precision diagnostics aims to improve patient management and outcome by stratifying patients for their risk of developing Acute Kidney Injury (AKI) and phenotyping kidney damage in the individual to enable tailored treatment [1, 2]. To benefit from this potential, unmet clinical needs should drive test development to truly improve clinical care pathways.

In general, the development of promising biomarkers to useful medical tests is a laborious and tedious process. Moreover, it is uncertain as the clinical, operational and the economic impact of a new test (panel) cannot directly be assessed during the translational phase from research to local clinical practice [3]. A framework for medical test evaluation has been established by the European Federation of Clinical Chemistry and Laboratory Medicine (EFLM) Working Group (WG) on Test Evaluation (TE) to guide researchers, laboratory specialists and clinicians during this process [4]. The TE framework considers the dynamic interrelation between unmet clinical needs, the clinical pathway, the analytical and clinical performance, the clinical and cost-effectiveness and the broader impact of medical tests. Mapping of the clinical care pathway(s) and predefining analytical (APS) and clinical performance specifications (CPS) are essential steps for test evaluation [5, 6]. Once the clinical care gaps have been identified, the biomarker selection process can commence. This specific approach, driven by unmet clinical needs, has not yet been applied to kidney injury.

In the case of kidney injury, the term AKI is used to indicate an abrupt (within hours) decrease in kidney function, which encompasses both structural damage (renal AKI) and loss of function without structural damage (prerenal AKI) [7, 8]. The latest classification of Acute Kidney Injury proposed by the Acute Kidney Injury Working Group of KDIGO (Kidney Disease: Improving Global Outcomes) defines AKI based on the renal function parameters urine output (i.e. urine output < 0.5 ml/kg/h for 6 h) and serum creatinine (i.e. increase ≥ 26.5 µmol/l within 48 h) and subdivides the severity of AKI into three stages based on the same parameters and Renal Replacement Therapy (RRT) is added to the definition of stage three [17]. AKI is a syndrome with a broad spectrum of causes and pathophysiologies and the functional parameters creatinine and urine output that are used to define and diagnose AKI cannot distinguish between prerenal AKI due to a drop in glomerular filtration pressure, and renal AKI [7, 9]. Furthermore, these parameters poorly represent early kidney damage, as serum creatinine only increases once the renal reserve capacity is exceeded. Therefore mild or early kidney damage frequently remains unnoticed [1]. It is highly likely that a loss of 25% of kidney function or 25–30 ml/min per 1.73 m^2^ of GFR in a patient with normal baseline function will be undetectable by serum creatinine [9]. While kidney function markers have proven useful for the clinical definition of AKI, they lack specificity towards kidney damage and its potential etiologies [10]. Given the large burden on individual patient health and the healthcare system, a more timely diagnosis of renal AKI and of the anatomical site of damage and of the underlying cause is needed. A multi-marker test could potentially fulfil this clinical need and enable a precision medicine approach.

**Fig. 1:**
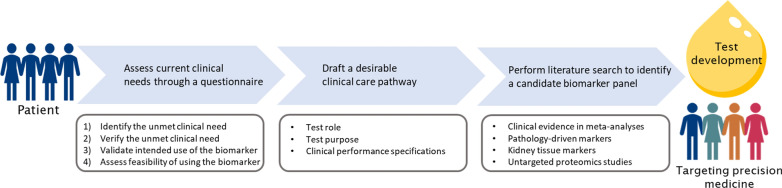
Strategy for the rational biomarker selection and test development driven by unmet clinical needs in kidney injury. Clinical needs were identified by nephrologists using a peer reviewed EFLM Test Evaluation questionnaire. Subsequently, desirable test roles, test purposes and clinical performance specifications in the clinical pathway were defined. Through a literature study a candidate biomarker panel is proposed that could meet existing gaps in current practice and aims to improve clinical practice and outcome. A multiplex test is in development to enable precision diagnostics in kidney injury

In this study we pilot the EFLM unmet clinical needs questionnaire for kidney injury biomarkers and evaluated kidney care pathways with nephrologists to identify existing clinical gaps in contemporary test-treatment pathways at the Leiden University Medical Center (Fig. 1). After identifying the clinical needs and drafting the desirable performance characteristics, biomarkers that theoretically have the potential to close the gaps were extracted either (A) from meta-analyses examining the clinical performance in kidney injury prediction, (B) from pathology-driven hypotheses, (C) from kidney tissue protein expression data and (D) from untargeted proteomics studies. Finally, we propose a multiplexed biomarker panel for a lab-developed test that has the potential to meet the four clinical gap categories.

## Assessment of clinical care gaps in patients with kidney injury

To aid effective translation of biomarkers to medical tests, the EFLM Working Group on Test Evaluation developed a structured questionnaire to identify and verify unmet clinical needs, to validate the intended use, to assess the feasibility of the new test (panel) and its impact on clinical practice and health outcome [11, 12]. This questionnaire consists of four steps: (1) identification of the unmet clinical needs in current practice, (2) discussion of potential solutions, (3) validation of the intended use and (4) assessment of the feasibility of applying the new test [11, 12]. In this study, nephrologists at the Leiden University Medical Center (LUMC), an academic center with expertise in Transplantation and Immunity, Oncology and Regenerative Medicine, were invited to pilot this structured questionnaire (Additional file 1). In response to a formal introduction on the EFLM unmet clinical need questionnaire, eighteen clinical needs were formulated by seven nephrologists and these responses were grouped into four key unmet clinical needs for kidney injury testing. Below we focus on steps one and two of the questionnaire: the identification and verification of the clinical needs.

### Existing clinical care pathways

Currently, clinicians mainly rely on markers for the glomerular filtration rate (GFR), markers for the combined effect of GFR and tubular function (urine output and fractional excretion of solutes) and markers signifying glomerular injury (proteinuria and glomerular hematuria), to diagnose kidney injury. When AKI is suspected, after ruling out a postrenal cause, fluid resuscitation to optimize volume status is the primary action to assess reversibility of kidney function. Non-responsiveness to fluid assessment may indicate renal AKI. In case a glomerular or tubular disease is considered likely, specific laboratory tests on blood (e.g. serology for auto-inflammatory diseases), imaging and a kidney biopsy are important tools to aid in making a diagnosis and to guide treatment [13]. In addition, tubular dysfunction can be recognized by increased renal excretion of low molecular weight proteins (e.g. β-2-microglobulin), presence of granular casts and renal tubular epithelial cells (RTECs) in the urine sediment and electrolyte abnormalities [14]. Despite the availability of these tests an unmet clinical need remains.

### Identification of the clinical gaps in the current clinical care pathways

To optimize patient care in the LUMC, we defined four major unmet clinical needs based on the nephrologists’ responses in the questionnaire. These needs are (1) early diagnosis of in-hospital AKI after a medical insult and in critically ill patients, (2) risk stratification for kidney injury prior to a scheduled (elective) intervention, (3) kidney injury monitoring in patients scheduled to receive nephrotoxic compounds and after kidney transplantation, (4) differentiation between prerenal AKI and structural kidney damage (Table 1).

**Table 1:** Unmet clinical needs and desirable biomarker characteristics and clinical performance specifications

Unmet clinical need	Target population	Key clinical endpoints	Desirable biomarker kinetics	Test purpose and test role	Desirable clinical performance specifications
Early diagnosis of in-hospital AKI after a medical insult and in critically ill patients	Critically ill patients At ICU admission After cardiothoracic surgery	AKI RRT ICU stay	Early rise and protracted fall kinetics	Prognostic marker Add-on testing	Improve detection rate kidney injury Sensitivity outweighs specificity NPV > 80–95%
Risk stratification for kidney injury prior to a scheduled intervention	Overall hospital population/at admission Patients with stable eGFR	AKI RRT CKD ICU stay hospital stay	Altered at baseline level	Prognostic marker Triage testing	Specificity outweighs sensitivity PPV > 80–95%
Patient monitoring for kidney injury and progression	Patients receiving nephrotoxic medication Kidney transplantation recipients	RRT CKD ESRD Allograft function/rejection	Relation between biomarker levels and damage	Monitoring marker Add-on testing	Specificity outweighs sensitivity PPV > 80–90% Increase before kidney function decline
Differentiation between prerenal AKI and structural kidney damage	Patients with suspected structural kidney injury Patients with established AKI	RRT duration CKD ATN, AIN ESRD	Kidney-topography specific biomarker release patterns	Diagnostic marker Add-on/replacement testing	Ruling out prerenal AKI and reduce unnecessary treatment in patients with prerenal AKI Acceptable sensitivity High specificity (> 85%)

First, timely diagnosis of kidney injury after an intervention such as cardiothoracic surgery and intensive care unit (ICU) admission is a clinical need. The sudden decline in kidney function is poorly predictable and occurs frequently in (critically) ill patients. In addition, patients with AKI may need temporary or continuous RRT and have an increased risk to develop CKD. Through early recognition of kidney injury, the incidence of progression to AKI, as defined by the KDIGO criteria, and need for RRT may be reduced [15].

Second, injury risk prediction prior to an intervention, including elective surgery or nephrotoxic medication in the general hospital population would provide patient benefit. Risk stratification for AKI is based on clinical risk factors, such as kidney function, medication and type of surgical or medical intervention. In practice, this stratification has been considered inadequate [16]. Biomarker-guided stratification of patients with stable kidney function into high and low AKI risk groups might enable differential therapies or dosing strategies and more stringent kidney function monitoring.

Kidney damage monitoring during and after exposure to nephrotoxic medication is a third unmet clinical need. Early and non-invasive detection of kidney damage could enable precision medicine by preemptive dose adjustments and therapy switches in response to the course of kidney damage markers. Non-invasive kidney damage monitoring would be beneficial for instance in patients receiving cytostatic agents, nephrotoxic antibiotics or calcineurin inhibitors. In clinical practice, it might be unclear whether a serum creatinine-based kidney function decline is a result of a medical treatment, comorbidities or underlying kidney disease. For example, when the kidney function decreases in kidney allograft recipients with calcineurin inhibitor therapy for immunosuppression, this decline may be due to an acute rejection episode or acute calcineurin inhibitor toxicity [17].

A fourth identified clinical need is the differentiation between prerenal AKI and structural damage with localization of affected tissue. Causes of AKI can be classified in either prerenal, renal or postrenal [18]. Prerenal AKI implies that the observed decline in urine output and creatinine clearance is primarily caused by alterations in the effective circulating volume, renal hypoperfusion and subsequently glomerular filtration (e.g. in bleeding, dehydration, sepsis syndrome and heart failure) [19]. For optimal and personalized treatment of AKI, there is a need to differentiate between primarily prerenal AKI and early structural ischemic renal damage, such as acute tubular necrosis (ATN) [2, 20]. In practice, biomarkers that reflect the transition of prerenal AKI to structural renal damage would be beneficial for patient management, for example, to guide fluid resuscitation in patients with unstable kidney function [21]. Biomarkers that could localize kidney damage in glomerular, tubular, interstitial and/or vascular compartments are desired. Since currently available laboratory parameters barely provide histological information, kidney biopsy remains needed for differential diagnosis of renal pathologies, such as ATN and acute interstitial nephritis (AIN) [14, 22–24]. To this end, an ideal biomarker panel should indicate the affected nephron compartments and provide insight into the underlying causes of sudden kidney function decline.

### Opportunities for optimizing contemporary clinical care pathways

In the second step of the EFLM checklist it is determined whether the development of a new test (panel) is justified. Therefore, alternative improvements of the clinical care pathway are discussed on their potential to reach similar objectives [12]. One potential solution could be to increase awareness among clinicians for AKI and related adverse clinical outcomes. Also, profound education on patient volume status assessment, the exposure of nephrotoxic medication in patients with high AKI risk and the necessity of stringent urine output monitoring and reporting, could likely reduce the incidence of AKI [25, 26].

A second alternative improvement could be the use of electronic health (eHealth) monitoring to longitudinally and actively assess currently available laboratory parameters. For example, eHealth or AKI alert systems are available to stratify individuals with increased risk for developing kidney injury [27, 28]. However, it is currently unsure whether AKI alert systems for inpatient management improve clinical outcomes [29]. In CKD patients or kidney transplant recipients, self-monitoring of kidney function by eHealth allows efficient and cost-effective outpatient disease management [30, 31].

Improvement of conventional urinalysis is a third alternative solution [14]. Extension of urine sediment analysis to include specifics on dysmorphic erythrocytes, pathological casts and renal tubular epithelial cells (RTECs) could aid the differentiation between AKI with prerenal cause and different types of structural renal damage (e.g. ATN, AIN, nephritic syndrome and nephrotic syndrome) [24, 32–35]. Nowadays, fast and standardized automated urine sediment analysis may be achieved by state-of-the-art urine analyzers, but specificity for nephrological structures remains too limited and often still requires manual microscopic evaluation [36–38]. All these proposed strategies to improve outcomes in kidney injury may refine current clinical care pathways, but would not be sufficient in addressing the unmet clinical needs in kidney injury.

## Mapping the desirable clinical care pathway

**Fig. 2:**
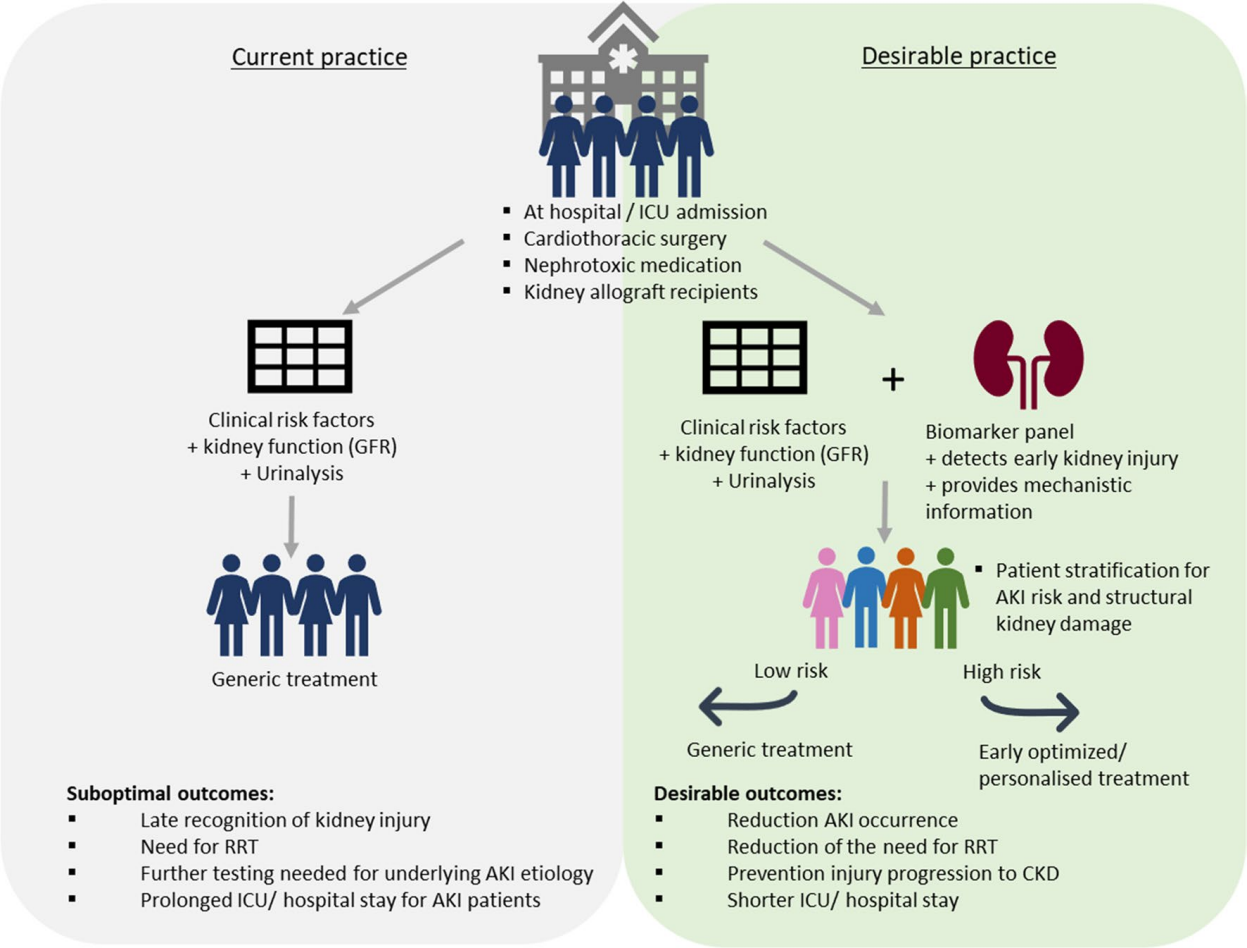
Paradigm shift from current practice to desirable clinical practice by targeting suboptimal detection of kidney injury using a kidney injury biomarker panel. Test purposes and test roles of individual panel proteins in the clinical care pathway are driven by the identified unmet clinical needs. Early optimised treatment may prevent conversion to irreversible structural kidney damage and would improve patient outcome

In the third step of the EFLM questionnaire the intended use of a novel biomarker test panel is validated by re-mapping the clinical pathway and discussing the envisioned impact on patient management decisions and health outcome [12]. A new kidney injury test should contribute to improved health outcomes and, therefore, the desirable biomarker characteristics and clinical performance specifications (CPS) should be predefined. Figure 2 illustrates the envisioned clinical pathway with the introduction of an add-on kidney injury protein panel aiming to improve patient outcome by early optimized personalized treatment.

### Desirable kidney injury biomarker characteristics

**Fig. 3:**
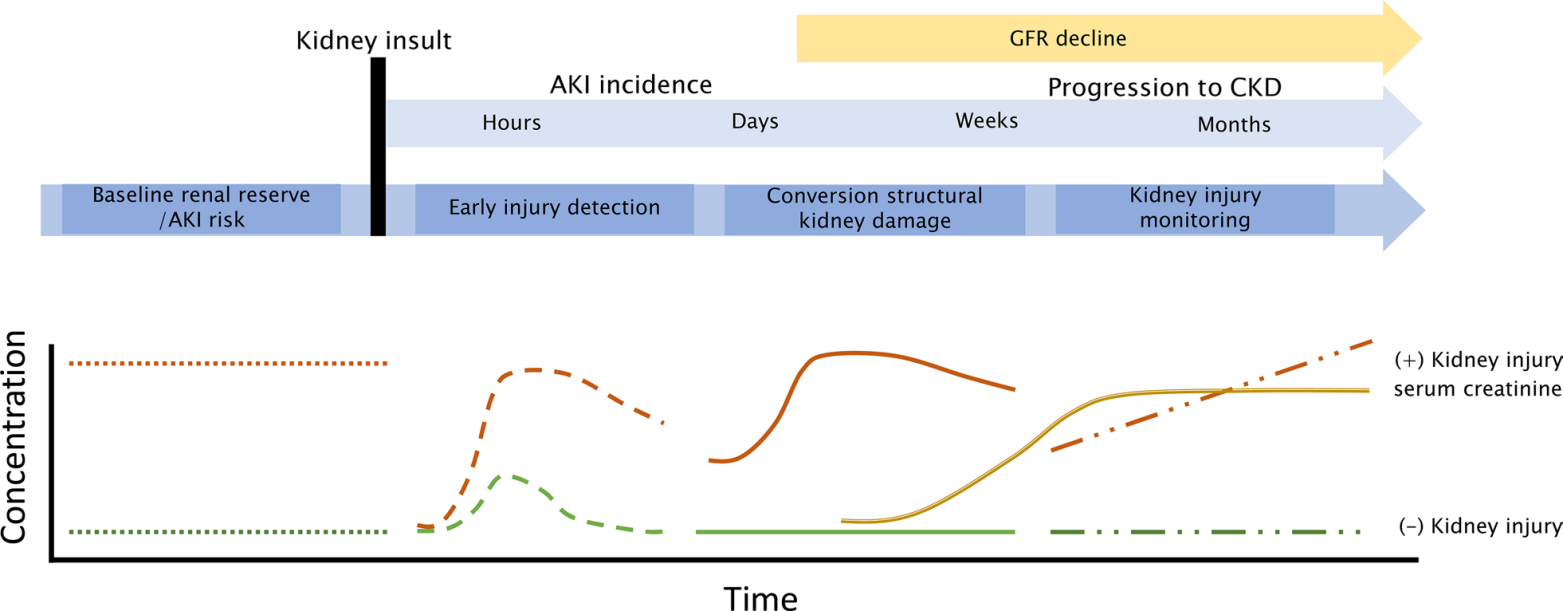
Desirable time kinetics of kidney injury biomarkers. The four unmet clinical needs in kidney injury all require specific biomarker rise and fall patterns. For early diagnosis, early rises within hours are essential whereas for late diagnosis a protracted time kinetic is needed. For risk stratification the biomarker concentration should be altered prior to the intervention. For kidney injury monitoring, a close relation to structural damage is needed

Biomarker kinetics should reflect the intended use of a biomarker, such as early detection of kidney injury prior to or directly after a scheduled medical intervention (Fig. 3). For effective patient management in the critically ill, kidney injury test results need to be available directly after an intervention or ICU admission. Indeed, the AKI prediction marker [TIMP-2]*[IGFBP7] is marketed as point-of-care test and its concentration-based output rapidly increases in response to injury and peaks within 12 h after the insult [39]. The timing of urine specimen collection is pivotal for AKI prediction after an intervention, because it strongly affects test performance [39, 40].

For patient stratification prior to an intervention, a biomarker should have an altered concentration at baseline to be meaningful in clinical decision making. In kidney injury monitoring biomarkers in sequentially collected urine specimens should reflect stagnation or progression of damage. For kidney injury differentiation, a biomarker (panel) ideally indicates the injured nephron compartment and reflects pathological lesions seen on biopsy, such as ATN [1, 10, 34].

### Desirable clinical performance specifications of kidney injury test(s)

For the development of a clinical test, the purpose and role should be specified, because the Clinical Performance Specifications (CPS) depend on its intended use [5]. The test purpose describes the intended clinical application (e.g. prognosis, diagnosis or monitoring) and the test role indicates the test position in the clinical pathway (e.g. add-on, triage or replacement). The test role and purpose vary between the four clinical needs defined here, as outlined in Table 1.

For early recognition of AKI after an intervention, a suitable biomarker should improve the detection rate of kidney injury, ideally by timely elevations ahead of serum creatinine rises. To achieve such clinical performance a cut-off value resulting in better sensitivity than specificity should be set (desirable negative predictive value (NPV) > 80–95%). The early diagnosis of kidney damage should induce preventive measures to reduce progression to AKI. Desirable health outcomes are the reduction in RRT incidence and ICU stay [41, 42]. Although early kidney injury detection enables early treatment, effective interventions that show improved clinical outcome after early biomarker-guided injury detection remain limited [43, 44]. In critically ill patients, the potential benefits of reducing kidney injury-related complications are likely to outweigh the harms accompanied by excessive patient monitoring, such as associated health care costs.

Prognostic markers are needed to classify the risk for developing AKI with the need for RRT, CKD and end-stage renal disease (ESRD). Patient stratification for these risks, should be applicable to the overall hospital population before any scheduled elective intervention with AKI-inducing adverse effects. To minimize unnecessary adjustments in scheduled treatments of non-critically ill patients, the specificity should outweigh the sensitivity in this test role.

In patient monitoring, add-on testing would ideally guide therapy by initiating, discontinuing or adjusting a medical treatment. For instance, in a transplantation setting with patients receiving calcineurin inhibitors for immunosuppression, potential nephrotoxicity may be monitored with kidney damage markers in addition to therapeutic drug monitoring [17, 45]. Such markers may aid the monitoring for (acute) kidney allograft rejection, aiming for the prevention of progressive fibrosis and (early) graft loss [46–48].

For the differentiation of prerenal AKI and structural kidney damage, a useful test (panel) should discriminate ATN from other clinical conditions and comorbidities that affect urinary output and serum creatinine [21]. Subsequently, such a test should preferably have a high specificity to rule out patients with transient AKI with prerenal etiology that can be restored by optimization of the effective circulating volume by fluid resuscitation- from AKI with structural renal damage. E.g. urine sediment analysis may aid the recognition of ATN or AIN after AKI risk stratification by a marker with lower specificity for structural damage [49].

## Literature search strategy to select biomarkers that address the clinical needs in kidney injury testing

Multiple biomarkers are needed to address the different clinical care gaps for kidney injury assessment. Four literature search strategies were applied for the selection of candidate protein-based biomarkers in urine (Additional file 2: Table S2.1). First, biomarkers were selected based on clinical evidence for kidney injury prediction and their association with AKI and RRT. Subsequently, biomarkers were extracted from previously proposed biology-driven hypotheses in renal pathologies. In the third strategy proteins with enhanced expression within the kidney and in specific nephron compartments were identified from the Human Protein Atlas (https://www.proteinatlas.org/). Finally, untargeted proteomics studies were discussed to identify promising alternative biomarkers for kidney injury.

### Biomarkers from clinical evidence in meta-analyses

Evidence of clinical performance for the prediction of AKI, AKI severity and RRT in critically ill patients was obtained from meta-analyses. Meta-analyses were available for the urinary biomarkers kidney injury molecule-1 (KIM-1) [50], neutrophil gelatinase-associated lipocalin (NGAL) [51–56], interleukin-18 (IL-18) [52, 54, 57, 58], *N*-acetyl-β-d-glucosaminidase (NAG) [54], cystatin C [52, 54, 59], liver-type fatty acid binding protein (L-FABP) [54, 60], metalloproteinase-2 (TIMP-2) and insulin-like growth factor-binding protein 7 (IGFBP7) [52, 61–64] (Additional file 2: Table S2.2).

Two of the meta-analyses compared two or more urinary kidney injury biomarkers [52, 54]. Urinary NGAL, KIM-1, L-FABP, IL-18, NAG and cystatin C demonstrate modest discriminative performance (AUCs < 0.75 for NAG and cystatin C, and < 0.70 for KIM-1, NGAL, IL-18 and L-FABP) for AKI prediction within 24 h after cardiac surgery [54]. Urinary cystatin C, IL-18, NGAL and the product of TIMP-2 and IGFBP7 were also evaluated for the prediction of RRT in critically ill patients [52]. The product TIMP-2 and IGFBP7 yielded the best predictive value (AUC = 0.86) and urinary cystatin C was the second best performing biomarker (AUC = 0.79). The largest body of evidence was available for NGAL with an AUC of 0.72 (n = 17) [52].

### Candidate pathology-driven biomarkers

Kidney injury is a multifactorial syndrome with multiple underlying pathologies (Additional file 2: Table S2.3). Insults that induce renal ischemia or direct cytotoxicity are usually the stimuli for AKI occurrence. Individuals with underlying kidney damage or disease are more susceptible to develop acute complications [65–67].

Hospital-acquired renal ischemia or ischemia–reperfusion injury (IRI) is typically procedure-related and occurs after cardiothoracic surgery with cardiopulmonary bypass or organ transplantation. In ischemic conditions, the complement system is activated and (pro) inflammatory cytokines and chemokines are released [68–71]. For instance, depositions of complement factors C3, C6, C9 and mannose-binding lectin (MBL) were found in ischemic kidneys [72], and elevations in systemic and urinary levels of chemokines CXCL9 and CXCL10 have been procedure-related ischemia and acute renal allograft rejection [70]. In addition, these chemokines, and in particular CXCL9, have been proposed as noninvasive markers of IRI induced renal allograft rejection [71, 73].

Ischemia may also induce structural kidney injury in the proximal tubules [74]. Tubular kidney damage may be characterized by histology-based kidney classification, such as ATN and tubulointerstitial nephritis (TIN). These are pathologies typically seen after exposure to medication with direct renal cytotoxicity are TIN and acute interstitial nephritis (AIN). Urinary IL-18, NGAL, KIM-1, L-FABP and albumin have been proposed as biomarkers for ATN, but their specificity for this structural pathology remains limited [75, 76]. Damage to the renal tubules impairs the reabsorption of filtered ions, metabolites and low molecular weight proteins resulting in an increased fractional excretion [14]. Therefore, the concentration of low molecular weight proteins, such as β-2-microglobulin (14 kDa), retinol-binding protein (16 kDa) and cystatin C (16 kDa), reflects tubular reabsorption functioning [77]. The bone-derived hormone FGF-23 inhibits tubular phosphate transport and has been proposed as marker of CKD [78, 79].

Individuals with pre-existing kidney damage or CKD are at increased risk for AKI. Both conditions are characterized by increased permeability of the glomerular filtration barrier and ultimately leading to proteinuria and hematuria. This is caused by podocyte detachment from the glomerular slit diaphragm [80]. The proteins podocin and nephrin play a role in maintaining the slit diaphragm and are candidate biomarkers of early glomerular damage [81]. Other candidate mechanistic markers could be podocalyxin [82, 83], the main protein in the glomerular glycocalyx, which is involved in glycocalyx degradation [82, 84].

Later stage CKD may be characterized by fibrosis, in which the extracellular matrix is reorganized [85]. Current fibrotic markers for CKD progression include transforming growth factor beta-1 (TGF-β1), monocyte chemoattractant protein 1 (MCP-1) and metalloproteinase (MMP) 2 [86], as well as a 273 peptide panel [87, 88]. In a recent study, the proteins chitinase 3-like protein 1, growth hormone 1 and MMP2, MMP7, MMP8, MMP13, tyrosine kinase and tumor necrosis factor 1 were validated as a biomarker panel for GFR prediction in CKD [89].

### Kidney topography markers

AKI biomarkers KIM-1, NGAL, TIMP-2 and IGFBP7, are widely expressed through the human body, including the proximal and/or distal tubules in the kidneys [90, 91]. Tissue selective proteins could provide anatomical information in kidney injury. Proteins that are specific for or enriched in glomeruli, proximal/distal tubules, the loop of Henle and the collecting duct were identified as candidate biomarkers using The Human Protein Atlas (Additional file 2: Table S2.4).

Within the glomeruli, podocin, nephrin and nephrin-like protein 1 are highly abundant and expressed on the surface of podocytes [92, 93]. Of these proteins, nephrin and podocin have already been proposed as early biomarkers for kidney diseases, such as diabetic nephropathy [94, 95].

In the proximal tubules, transporter proteins from the solute carrier superfamily (SLC) are expressed at epithelial linings. Interestingly, variants in the genes coding for SLC22A2 and SLC22A12 were related to susceptibility for kidney disease [96, 97], and SLC22A2 polymorphisms are related to maintenance of kidney function after cisplatin exposure [98]. Two other proteins in the proximal tubules are the transporters cubilin and megalin, which together facilitate the reabsorption of proteins filtered by the glomeruli (e.g. cystatin C and NGAL) [77]. Cubilin and megalin have been evaluated as markers for Fabry disease [99].

In the distal tubules SLC12A1, SLC13A3, calbindin and uromodulin (Tamm-Horsfall glycoprotein) are typically enriched [92]. Of these proteins, calbindin, which is a member of the calcium-binding protein superfamily, has been proposed as biomarker for early kidney injury detection after treatments with cisplatin [100]. Uromodulin is exclusively produced by tubular cells and has been proposed as measure of the total functional nephron mass to stratify patients with mild CKD for their risk of progressive disease and ESRD [101–103]. A smaller total functional nephron mass may reveal kidney injury susceptibility, which could explain why lower preoperative uromodulin levels were found associated with AKI development after cardiac surgery [104]. Moreover, genome-wide association studies have identified several uromodulin common variants that are associated with higher GFR and lower risk of CKD [97, 105].

### Kidney injury biomarkers identified in untargeted urine proteomics studies

Untargeted proteomics, is a powerful tool to discover novel biomarkers that are associated with a state of disease [106]. Clinical proteomics studies can provide insight into molecular pathways in kidney injury. Currently, eight biomarker discovery studies address the human proteome in kidney injury using an untargeted approach (Additional file 2: Table S2.5). Of the four unmet clinical needs identified in this study, risk stratification prior to a medical intervention remains poorly addressed with the so far identified markers.

To address this need, we focused on the proteomics studies in which the clinical endpoint AKI was defined [107–111]. Interestingly, one of these studies looked into pre-operative kidney injury biomarkers and found that CFB and HRG were associated with post-surgery AKI risk and enhanced the performance of conventional clinical risk scoring tools [107]. In another study, the urine proteome before and after CPB was compared and altered levels were found of inflammation-associated ZAG, LRG, MASP2, HSPG, and IGKV1-5 and tubular dysfunction proteins uromodulin, RBP and AMBP [108]. Although the exact role of these proteins in kidney injury remain to be unraveled, the involvement of immune-related proteins seems evident. A protein panel ideally differentiates between injury pathologies, as has previously been demonstrated in kidney allograft recipients [112]. Although multiple urinary proteins have been found to be associated with kidney injury in untargeted proteomics studies, these candidate markers remain to be clinically validated for the diverse clinical conditions that occur in an hospital setting.

### A theoretical biomarker panel for kidney injury

**Fig. 4:**
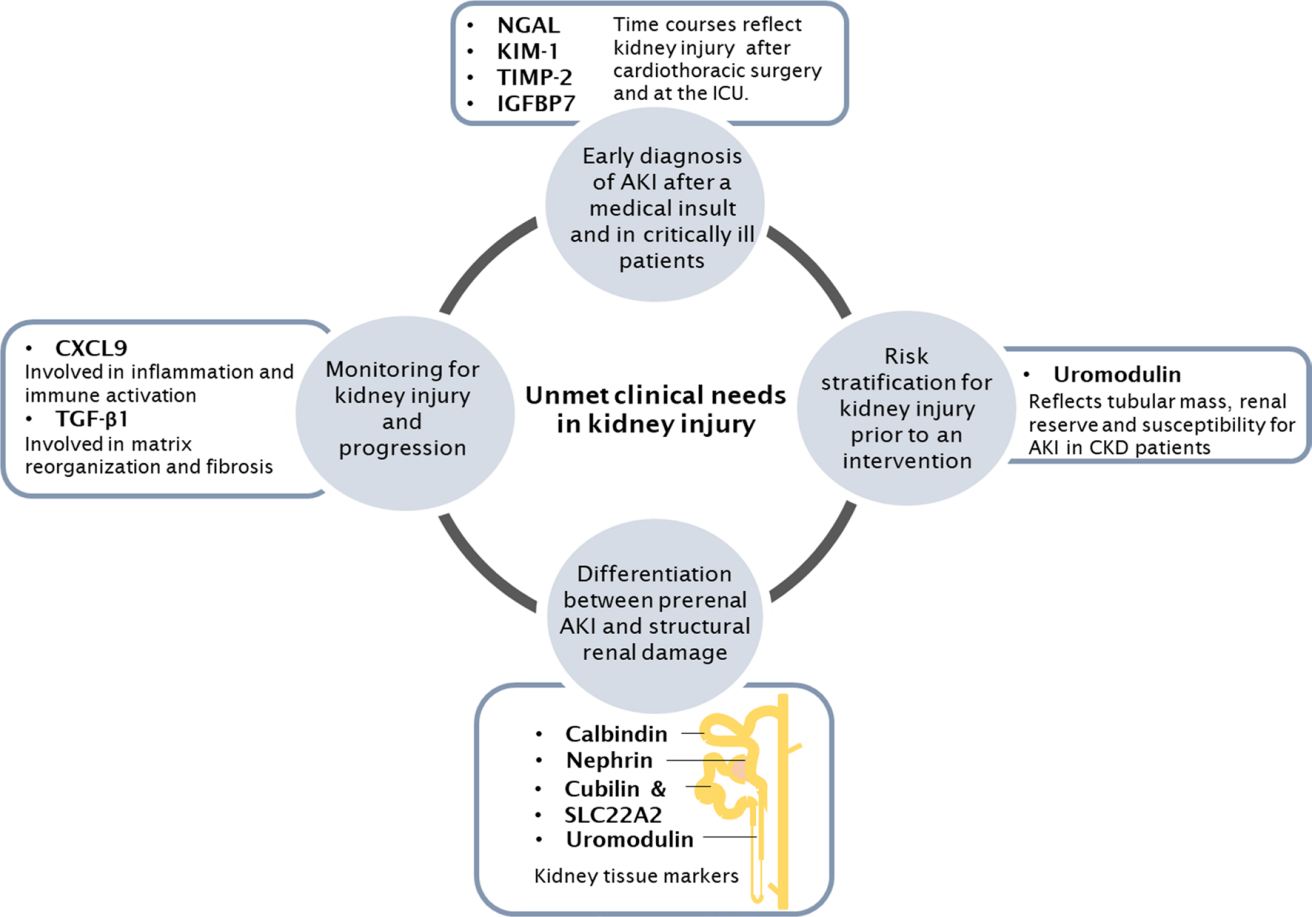
Proposed kidney injury biomarker panel targeting the unmet clinical needs in kidney injury at the Departments of Nephrology and Clinical and Laboratory Medicine, Leiden, The Netherlands. Four major clinical gaps were identified in kidney injury testing using a questionnaire. After verification of the needs, a literature search was performed and eleven candidate biomarkers were selected for a mass spectrometry-based test to address the unmet clinical needs

In the follow-up of the literature study of biomarker candidates, proteins were selected for inclusion in a multiplex lab-developed test. The composition of the biomarker panel was based on potential to address all four identified unmet clinical needs. To maximize this potential, both clinically relevant and biology-driven biomarkers, often with yet unknown clinical relevance, were combined. The unmet clinical need for early diagnosis of in-hospital AKI after a medical intervention and in critically ill patients (clinical need I) could be targeted by well-studied early injury markers described in (paragraph “Biomarkers from clinical evidence in meta-analyses”). Considering the commercial availability of cystatin C test on routine chemical analyzers, TIMP-2, NGAL, KIM-1 and IGFBP7 were selected for inclusion in a mass-spectrometry-based test panel. To our knowledge, there is poor clinical evidence for biomarker-guided risk stratification prior to an intervention, such as major surgery or ICU admission (clinical need II). To this end, urinary uromodulin, which has previously been described for the assessment of baseline injury risk [102], was added to the biomarker panel to evaluate its potential for translation toward clinical practice. The clinical need for kidney injury monitoring after organ transplantation or exposure to nephrotoxic compounds (clinical need III), will be targeted by CXCL9 as marker for ischemia-induced allograft rejection, and TGF-β1 to indicate tissue fibrosis in injury progression. Nephron compartment-enriched proteins nephrin (glomerulus), SLC22A2, cubilin (proximal tubule), calbindin (distal tubule) and uromodulin (distal tubule & Loop of Henle) were selected to potentially address the need for the differentiation between prerenal AKI and ATN (clinical need IV) and facilitate localization of kidney damage. Figure 4 illustrates the proposed hypothesis-driven protein biomarker panel for translation research.

In the medical laboratory, proteins are commonly quantified indirectly by automated immunoassays. However, the development of specific and sensitive immunoassays is tedious and costly, often in uniplex test formats, and these tests are prone to several types of interferences [113, 114]. Mass spectrometry has been proposed as an alternative for multiplex protein quantitation in the clinical chemistry laboratory [114]. Liquid chromatography (LC) coupled to multiple reaction monitoring (MRM) MS allows rather “fast” method development and multiplex protein quantitation with high analytical selectivity and sensitivity [115, 116]. Recently our laboratory was able to show reproducible absolute protein quantitation with LC-MRM-MS within and across laboratories [116–118], and long-term stability of test results was achieved through stringent quality control and instrument performance monitoring [117]. Multiplex LC-MRM-MS technology may be the preferred analytical methodology for setting up test applications that enables molecular characterization of proteins and efficient multiplex evaluation of biomarkers in the translational pipeline.

A multiplex mass spectrometry-based lab-developed test is currently in development to assess its analytical and clinical performance of the here proposed biomarker panel. The panel will be compared to conventional markers, such as urine sediment analysis, osmolality, albumin and tubular dysfunction markers β-2-microglobulin and cystatin C in urine [119, 120]. Effective clinical evaluation will enable the translation of our promising candidate biomarker panel toward clinical practice and potentially directly improve clinical care pathways for the benefit of patients. While rapid performing platforms, such as immunoassays on automated chemical analyzers or point-of-care devices, are required for clinical utility of routine AKI patient management in the acute setting, LC-MRM-MS may be the preferred tool for in-depth biomarker translational research.

## Conclusion

Medical test development is ideally driven by clinical needs in clinical care pathways, rather than by technological push. We here describe a first pilot experience with a structured translational approach to identify and verify gaps in clinical care pathways that encounter kidney injury burden. Four major clinical needs were identified by nephrologists in our academic centre (Fig. 4). To fill in these clinical gaps, promising biomarkers were selected from literature based on clinical evidence and biology-driven hypotheses. Due to the complex and multifactorial etiology of kidney injury and the risk of progression and other sequalae, a multi-test approach that allows precision diagnostics was preferred. Crosstalk and discussions between nephrologists, lab specialists and researchers were needed to explain the unmet clinical need checklist and to guide the process of identifying opportunities to improve existing clinical care pathways in patients with (risk of) kidney disease. In our hands, the EFLM unmet needs questionnaire has been experienced as a valuable tool as the checklist helps to structure the dialogue between clinicians and laboratorians, to reflect on the intended use of biomarkers in the clinical pathway and to rationalize the envisioned selection and use of medical tests in care pathways ahead of doing any clinical evaluation. Upon identification of the unmet clinical needs, the analytical and clinical performance specifications, a biomarker panel had to be selected. Here, a rational and theoretical biomarker selection process was employed. It should be noted, that often more than one marker could be identified to address a specific need; we aimed to select those markers with the highest level of confidence. This was especially the case for tissue-enriched markers, that were selected mainly based on their kidney/tissue localization or role in kidney pathophysiology. Therefore, the clinical relevance of the proposed kidney injury biomarkers are now studied by multiplexed LC–MS analysis. To conclude, the proposed translational approach, in which clinical gaps in clinical pathways are identified using the EFLM checklist, and subsequently addressed with a rationally designed biomarker panel seems feasible. “Fast” evaluation of these markers using LC-MRM-MS based test should now reveal whether the proposed biomarker panel is clinically effective and has the potential to improve diagnostic stewardship for the sake of precision medicine.

## Supplementary Information

**Additional file 1.** EFLM unmet clinical needs checklist.

**Additional file 2: Table S2.1.** Literature search strategy biomarkers for kidney injury. **Table S2.2.** Collected meta-analyses. **Table S2.3.** List of biology-driven biomarkers. **Table S2.4.** Kidney tissue-enriched proteins. **Table S2.5.** Collected untargeted proteomics studies identifying kidney injury biomarkers in urine.

## Abbreviations

AIN: Acute interstitial nephritis
AKI: Acute kidney injury
ATN: Acute tubular necrosis
CKD: Chronic kidney disease
CPS: Clinical performance specifications
EFLM WG-TE: European Federation of Clinical Chemistry and Laboratory Medicine Test Evaluation Working Group
ESRD: End-stage renal disease
GFR: Glomerular filtration rate
ICU: Intensive care unit
IRI: Ischemia–reperfusion injury
KDIGO: Kidney Disease Improving Global Outcomes
NPV: Negative predictive value (number of true negatives / number of negative calls)
PPV: Positive predictive value (number of true positives / number of positive calls)
RRT: Renal replacement therapy
TIN: Tubulo-interstitial nephritis
