# Supplementary material for: Rational selection of a biomarker panel targeting unmet clinical needs in kidney injury
Source: Clin Proteomics. 2021 Feb 22;18:10. doi: 10.1186/s12014-021-09315-z (PMC7898424; doi:10.1186/s12014-021-09315-z)
Supplement: Supplementary file 1 — Additional file 1. EFLM unmet clinical needs checklist. [file 12014_2021_9315_MOESM1_ESM.docx]

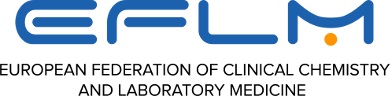


**Unmet clinical needs – the EFLM checklist**

Tutorial and digital version: <https://elearning.eflm.eu/course/view.php?id=11>

# **STEP 1: IDENTIFY THE UNMET CLINICAL NEED FOR A BIOMARKER**

## **Key question:** **What is the clinical management problem and desired outcomes?**

### **What is the health condition and clinical management problem?**

### **What is the target group?**

#### What patient groups are involved (symptomatic, asymptomatic)?

#### What is the setting?

### **What is current practice?**

#### Display current tests, clinical assessment tools and management decisions on clinical pathway (map the pathway to illustrate the intended use of new biomarker – (use ‘Mapping 1’ template provided below).

#### What data are available about the outcomes of current practice?

### **What are the limitations of current practice?**

#### What is the potential adverse or suboptimal outcome of the present pathway?

#### What are the health management gaps?

### **What are the desired outcomes?**

**STEP 2: VERIFY THE UNMET NEED FOR THE BIOMARKER**

**Key question: Is there an existing solution?**

**Could the problem be solved by:**

- *Optimising current practice*
- *Alternative clinical pathways/practices*
- *Implementation of evidence-based guidelines*
- *Quality improvement activities?*
- *Other (please specify):*

**Could these solutions be effective?**

**Could these solutions be cost-effective?**

**Are there any barriers for these solutions?**

**STEP 3: VALIDATE THE INTENDED USE OF THE BIOMARKER**

**Key question: Would the biomarker contribute to the solution?**

**How could the biomarker alter and improve current practice?**

Re-map the clinical pathway (*see ‘Mapping 2’ template* provided below) to show the intended use of the biomarker and proposed impact on management decision

**What are the expected outcomes of test results?**

- - *What are the positive or negative findings: benefits and harms?*

**How do these outcomes compare to the desired outcomes defined in Step1?**

- - *Are the trade-offs between benefits and harms potentially favorable?*
  - *What are the minimum clinical performance characteristics of the test for the trade-off to be favorable?*

**STEP 4: ASSESS FEASIBILITY OF USING THE BIOMARKER**

**Key question: Is the biomarker solution feasible in practice?**

**Under what conditions would the new biomarker solution be feasible?**

- *commercially (IVD industry)*
- *economically (health care organizations)*
- *technically (e.g. capital investment, pre-analytical, analytical performance, standardization)*
- *organizationally (e.g. facilities, patient flow, staff training, patient and stakeholder acceptability, accessibility, required availability– e.g. 24/7 lab service)*

**Are there any other barriers?**

(e.g. societal, ethical, legal aspects of care and impact on family members of the patient).

**CLINICAL PATHWAY MAPPING (CHECKLIST STEP 1**)

**Target population & setting**

(Primary care [GP, community], secondary, tertiary care,)

Country/health care system

☐ Primary care

☐ Secondary care

☐ Tertiary care

□Prognosis

Click here to enter text.

**New Biomarker intended use (role):**

Replacement

Add-on

Triage

Other (please specify)

**Current test(s) and clinical assessment tools**

Diagnosis

Prognosis

Screening

Prediction

Other (please specify)

□ Monitoring

Click here to enter text.

**Management decision**

(Therapeutic intervention, other)

Test Positive: Test negative:

**Health Outcomes:** Potential benefits for patient/physician:

Mortality

QoL (pain,employment)

Other (Please specify) Potential harms for patient/physician:

**Unmet needs:**

**CLINICAL PATHWAY MAPPING (CHECKLIST STEP 3)**

Health Outcomes

Benefits:

Harms:

Unmet needs:

New Biomarker intended use (role):

Replacement

Add-on

Triage

Other (please specify)

Potential Harms

Potential Benefits

Target population & setting

Current test(s) and clinical assessment tools

Test Positive

Test Negative

Management decision

Test Negative

Test Positive

Management decision

Health Outcomes

**Current practice**

**Intended use of new biomarker**
