# Supplementary material for: Rational selection of a biomarker panel targeting unmet clinical needs in kidney injury
Source: Clin Proteomics. 2021 Feb 22;18:10. doi: 10.1186/s12014-021-09315-z (PMC7898424; doi:10.1186/s12014-021-09315-z)
Supplement: Supplementary file 2 — Additional file 2: Table S2.1. Literature search strategy biomarkers for kidney injury. Table S2.2. Collected meta-analyses. Table S2.3. List of biology-driven biomarkers. Table S2.4. Kidney tissue-enriched proteins. Table S2.5. Collected untargeted proteomics studies identifying kidney injury biomarkers in urine. [file 12014_2021_9315_MOESM2_ESM.docx]

**Table S2.1: Literature search strategy biomarkers for kidney injury**


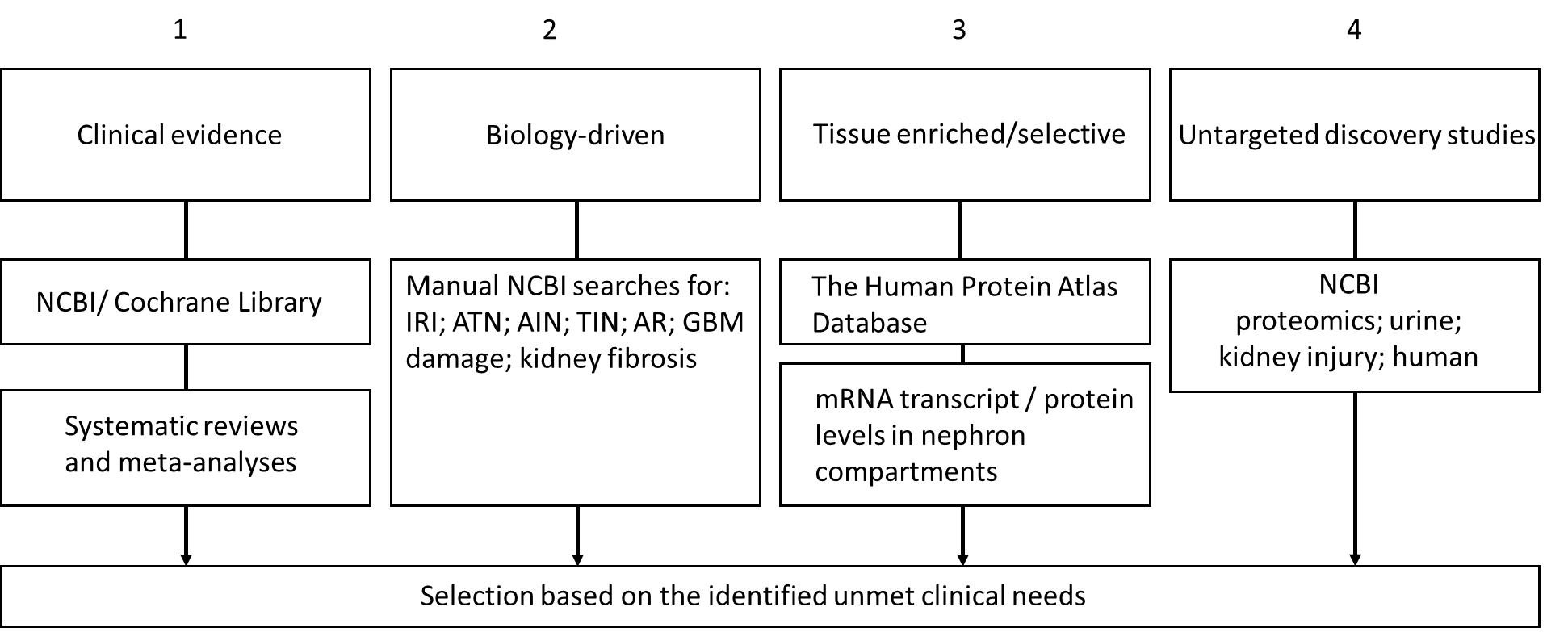


Figure S2.1 . Applied literature search strategies for candidate kidney injury biomarkers.

**Table S2.2:** **Collected meta-analyses from observational studies evaluating kidney injury biomarkers predicting AKI, AKI severity and/or need for RRT**

| Study | Clinical setting | Clinical endpoints | Evaluated biomarkers in urine |
| --- | --- | --- | --- |
| Zhang et al., Medicine (Baltimore). 2019; 98 | Critically ill patients | AKI | [TIMP2]*[IGFBP7] |
| Li et al., BMC Nephrol. 2019;20 | Kidney transplant recipients | DGF | NGAL |
| Klein et al., Intensive Care Med. 2018; 44 | Critically ill patients | RRT | NGAL, Cystatin C, IL-18, TIMP-2*IGFBP7 |
| Liu et al., Medicine (Baltimore). 2017; 96 | Critically ill adult patients | AKI | [TIMP2]*[IGFBP7] |
| Jia et al., Nephrology (Carlton). 2017; 22 | Critically ill patients | AKI, RRT | [TIMP2]*[IGFBP7] |
| Su et al., PLoS One 2017;12 | Critically ill patients | AKI, RRT | [TIMP2]*[IGFBP7] |
| Zhang et al., Crit Care. 2016;20 | Patients with sepsis | AKI, RRT, mortality | NGAL |
| Ho et al., Am J Kidney Dis. 2015; 66 | Adult patients undergoing cardiac surgery | AKI | NGAL, KIM-1, Cystatin C, NAG, L-FABP, IL-18 |
| Lin et al., J Nephrol. 2015;28 | Various clinical settings of AKI | AKI | IL-18 |
| Haase et al., J Am Coll Cardiol. 2011; 57 | Critically ill patients with cardiorenal syndrome | AKI, RRT, in-hospital mortality I | NGAL |
| Haase et al., Am J Kidney Dis. 2009;54 | Different clinical settings of AKI | AKI, RRT, in-hospital mortality | NGAL |
| Shao et al., PLoS One. 2014;9 | Various | AKI | KIM-1 |
| Susantitaphong et al., Am J Kidney Dis. 2013;61 | Various clinical settings of AKI | AKI, RRT,  In-hospital mortality | L-FABP |
| Liu et al., Am J Kidney Dis. 2013; 62 | Various clinical settings of AKI | AKI, in-hospital mortality | IL-18 |
| Zhang et al., Am J Kidney Dis. 2011; 58 | Various clinical settings of AKI | AKI | Cystatin C |

**Table S2.3: Collected biology-driven protein biomarkers involved in acute renal pathologies**

| **Type** | **Pathology** | **Biomarker** |
| --- | --- | --- |
| Overall/systemic | Inflammatory response to ischemia and cell death | - Complement activation: MBL(lectin), C3, C5a - Inflammatory cytokines and chemokines: IL-18, IL-6, YKL-40, TGF-β1, MCP-1 - CXCL9, CXCL10 |
|  | Fibrosis and matrix deposition | - TGF-β1, CTGF, PDGF, EFG, FGF-23, collagen fragments |
| Glomerular | Early damage glomerular basement membrane / podocyte detachment  Degradation of the glycocalyx | - Nephrin, podocin - Heparanase, podocalyxin |
|  | Increased glomerular permeability | - Total urinary protein, hematuria, urinary albumin, excretion HMW proteins (macroglobulin’s) - Dysmorphic erythrocytes in urine sediment |
|  | Deposition of immune-complexes in the glomeruli | - Immunoglobulins, complement factors |
| Tubular | Acute Tubular Necrosis | - Sediment analysis: muddy brown casts and RTECs (+) |
|  | Proximal tubular damage/ tubular dysfunction | - Tubular proteinuria, LMW proteins: Cystatin C, α1-microglobulin, β1-microglobulin, retinol binding protein |
|  | Distal tubular damage | - Tamm-Horsfall glycoprotein, π-Glutathione-S-transferase |
| Interstitial | Acute Interstitial Nephritis/Tubulointerstitial Nephritis | - Tubular proteinuria, LMW proteins: cystatin C, α1-microglobulin, β1-microglobulin, retinol binding protein - Inflammation markers: CRP - TGF-β1 |
| Vascular | e.g. bilateral vein thrombosis, bilateral renal artery emboli, renal infarction | - Imaging strategies: Ultrasound, contrast-enhanced CT |

**Table S2.4.1: List of top 12 genes with the highest level of enriched mRNA expression in the kidney**

| Gene | UniProt access number | Protein description | mRNA (kidney tissue) | Protein expression |
| --- | --- | --- | --- | --- |
| UMOD | P07911 | Uromodulin (Tamm Horsfall Glycoprotein) | 237.4 | Kidney specific (loop of Henle/ distal tubule) |
| SLC12A1 | Q13621 | Solute carrier family 12 member 1 | 143.2 | Kidney specific |
| MIOX | Q9UGB7 | Myo-inositol oxygenase | 136.7 | Kidney enriched |
| KCNJ1 | P48048 | Potassium voltage-gated channel subfamily J member 1 | 123.2 | In the kidney and pancreatic islets. Lower levels in skeletal muscle, pancreas, spleen, brain, heart and liver |
| FXYD4 | P59646 | FXYD domain-containing ion transport regulator 4 | 199.5 | Kidney enriched |
| SLC34A1 | SLC34A1 | Solute carrier family 34 member 1 (SLC17A2) | 94.5 | Kidney and lung |
| SLC22A12 | Q96S37 | Solute carrier family 22 member 12 | 70.1 | High expression in kidney (prox. tubules) |
| NPHS2 | Q9NP85 | NPGS2, podocin | 66.5 | Kidney specific |
| MCCD1 | P59942 | Mitochondrial coiled-coil domain protein 1 | 63.6 | Predominantly expressed in kidney |
| TMEM174 | Q8WUU8 | Transmembrane protein 174 | 61.5 | Predominantly expressed in kidney |
| TMEM207 | Q6UWW9 | Transmembrane protein 207 | 21.1 | Predominantly expressed in kidney |
| SLC6A18 | Q96N87 | Solute carrier family 6 member 18 | 14.1 | Predominantly expressed in kidney |

*Source: The human Protein Atlas (26-11-2019). ** mRNA≥ 14 fold change higher compared to tissue with second highest expression level ***Transcript profiling was based on a combination of three transcriptomics datasets (HPA, GTEx and FANTOM5, corresponding to a total of 483 samples from 37 different human normal tissue types.

**Table S2.4.2: Collection of renal compartment enriched proteins based on protein levels and localization with IHC**

| Compartment | Protein | Uniprot | Localisation |
| --- | --- | --- | --- |
| Glomerulus | Podocin | Q9NP85 | Podocytes; transmembrane |
|  | Nephrin | O60500 | Podocytes; transmembrane |
|  | KIRREL1/ Nephrin-like protein 1 | Q96J84 | Podocytes; transmembrane (also in placenta) |
| Proximal tubules | SLC22A8 | Q8TCC7 | Transmembrane; basolateral surface of epithelia |
|  | SLC22A13  SLC22A2 | Q9Y226  O15244 | Transmembrane; luminal surface of epithelia  Transmembrane; basolateral and luminal surface of epithelia |
|  | SLC28A1 | O00337 | Transmembrane; luminal surface of epithelia |
|  | SLC5A11 | Q8WWX8 | Transmembrane and intracellular (also some in the intestine and brain) |
|  | AGMAT | Q9BSE5 | Intracellular (also high expression in the liver) |
|  | BHMT | Q9H2M3 | Intracellular (also high expression in the liver) |
|  | Dihydropyrimidinase | Q14117 | Intracellular (also high expression in the liver) |
|  | Glutathione hydrolase 1 proenzyme | P19440 | Intracellular (also high expression in the epididymis, liver and pancreas. |
|  | RIDA | P52758 | Intracellular (also high expression in the liver) |
|  | LRP2/Megalin | P98164 | Intracellular and membrane (also high expression in parathyroid gland) |
|  | Cubilin | O60494 | Intracellular and membrane (also some in small intestine) |
|  | PKLR | P30613 | Intracellular (also high expression in the liver and hematopoietic cells) |
|  | X-prolyl aminopeptidase 2 | O43895 | Luminal surface renal tubules and intestinal glands |
| Distal tubules | Calbindin 1 | P05937 | Intracellular (vesicles) |
|  | SLC12A1 | Q13621 | Transmembrane; luminal surface of epithelia |
|  | SLC13A3 | Q8WWT9 | Transmembrane; basolateral surface of epithelia |
| Loop of Henle | Uromodulin | P07911 | Also distal tubule; intracellular, luminal surface of membrane and secreted |
| Collecting duct | Aquaporin-2 | P41181 | Transmembrane |
|  | V-type proton ATPase subunit d2 | Q8N8Y2 | Intracellular (vesicles) |
|  | Transmembrane protein 213 | A2RRL7 | Transmembrane and intracellular |

*Source: The human Protein Atlas (26-11-2019). Proteins visualised with IHC staining ** Habuka et al, PLoS One (2014); Fagerberg et al,
Mol Cell Proteomics (2014). Yu et al, Nucleic Acids Res (2015); Uhlén M et al, Sience (2015)

**Table S2.5: Collection of untargeted proteomics studies identifying kidney injury biomarkers in urine**

| Study | Approach | Population | Clinical endpoint | Technique |
| --- | --- | --- | --- | --- |
| Merchant et al., BMC Nephrol. 2018;19 | Untargeted | Patients that underwent cardiac surgery (pre-surgery) | AKI | LC-MS/MS Orbitrap |
| Aregger et al., Kidney Int. 2014; 85 | Untargeted | Critically ill patients | AKI | Gel electrophoresis and LC-ESI-MS/MS |
| Aregger et al., J Thorac Cardiovasc Surg; 2010; 139 | Untargeted | Patients that underwent CPB (proteome before and after CPB) | AKI | Gel electrophoresis and MALDI-TOF |
| Sigdel et al., Mol Cell Proteomics; 2014; 13 | Untargeted | Kidney transplant recipients | STA  AR  BKVN  CAN | LC-MS/MS Orbitrap |
| Devarajan et al., Am J Kidney Dis; 2010; 56 | Untargeted | Children that underwent CPB | AKI | SELDI-TOF |
| Ho et al., Am J Kidney Dis; 2009; 53 | Untargeted | Patients that underwent CPB | AKI | SELDI-TOF |
| Dwivedi et al., Clin Proteomics; 2016; 13 | Untargeted | Patients that underwent CPB (at start CPB and 1 -h into CPB) | - | LC-MS/MS TripleTOF |
| Vanhoutte et al., Nephrol Dial Transplant; 2007; 22 | Untargeted | CABG | - | 1) SELDI-TOF  2) 2D gel electrophoresis/  nano LC-MS/MS |

*STA = stable graft function; AR = Acute Rejection, BK = BK Virus Nephropathy, CAN = chronic allograft nephropathy
